# Rapid Mass Spectrometric Study of a Supercritical CO_2_-extract from Woody Liana *Schisandra chinensis* by HPLC-SPD-ESI-MS/MS

**DOI:** 10.3390/molecules25112689

**Published:** 2020-06-10

**Authors:** Mayya Razgonova, Alexander Zakharenko, Konstantin Pikula, Ekaterina Kim, Valery Chernyshev, Sezai Ercisli, Giancarlo Cravotto, Kirill Golokhvast

**Affiliations:** 1 REC Nanotechnology, School of Engineering, Far Eastern Federal University, Sukhanova 8, 690950 Vladivostok, Russia; zakharenko.am@dvfu.ru (A.Z.); k.golokhvast.ks@vir.nw.ru (K.P.); kim.emi@dvfu.ru (E.K.); chernyshev.vv@dvfu.ru (V.C.); golokhvast.ks@dvfu.ru (K.G.); 2 N.I. Vavilov All-Russian Institute of Plant Genetic Resources, B. Morskaya 42-44, 190000 Saint-Petersburg, Russia; 3 Agricultural Faculty, Department of Horticulture, Ataturk University, Erzurum 25240, Turkey; sercisli@gmail.com; 4 Dipartimento di Scienza e Technologia del Farmaco, University of Turin, Via P. Giuria 9, 10125 Turin, Italy; giancarlo.cravotto@unito.it; 5 Pacific Geographical Institute, Far Eastern Branch of the Russian Academy of Sciences, Radio 7, 690041 Vladivostok, Russia

**Keywords:** *Schisandra chinensis*, supercritical fluid extraction, HPLC-SPD-ESI-MS/MS, lignans

## Abstract

Woody liana *Schisandra chinensis* contains valuable lignans, which are phenylpropanoids with valuable biological activity. Among green and selective extraction methods, supercritical carbon dioxide (SC-CO_2_) was shown to be the method of choice for the recovery of these naturally occurring compounds. Carbon dioxide (CO_2_) was the solvent with the flow rate (10−25 g/min) with 2% ethanol as co-solvent. In this piece of work operative parameters and working conditions were optimized by experimenting with different pressures (200–400 bars) and temperatures (40–60 °C). The extraction time varied from 60 to 120 min. HPLC-SPD-ESI -MS/MS techniques were applied to detect target analytes. Twenty-six different lignans were identified in the S. *chinensis* SC-CO_2_ extracts.

## 1. Introduction

*Schisandra chinensis* (Turczaninowia) Baillon, is a medicinal plant (of the *Schisandraceae* family), known for its ethnomedicinal applications [1]. Its use in Chinese medicine dates back about 15 centuries, second only to ginseng. *S. chinensis* is included in the traditional Chinese medicine formula Sheng-Mai San, which has been used in the treatment of cardiovascular diseases [2]. *Schisandra*-based drugs, with the common names Shengmai-injection, Shengi Wuweizi-Pan and Shengmai-Yin are also included in the Chinese Pharmacopoeia [3].

The genus *Schisandra* (*Schisandraceae* family) consists of 25 species, two of which, namely *Schisandra chinensis* and *Schisandra repanda* (Maximowiczia nigra (Maxim.) Nakai), have a history of medicinal use [4]. *Schisandra chinensis* (synonyms: *Kadsura chinensis, Maximoviczia amurensis, Maximoviczia chinensis, Maximoviczia japonica, Sphaerostemma japonicum*, Wu wei zi) is endemic in northwest China (Heilongjiang Province), Korea, Russia (Primorye and Amur regions as well as Khabarovsk territory), Shikotan, Kunashir, Iturup, and on the island of Sakhalin.

Russian academician Komarov V.L. made a botanical description of *S. chinensis* (Turcz.) Baill. for the first time in 1901 and gave the first information about its healing effect, having brought it from expeditions to the Far East [5,6]. This is a deciduous liana, climbing up neighboring trees, up to 10−15 m long. The stem is covered with wrinkled, flaky dark brown bark. The leaves are elliptical 5−10 cm long, 3−5 cm wide, dicotyledonous flowers, up to 1.5 cm in diameter, with a distinct lemon aroma, multi-berry fruits, up to 10 cm long, juicy red seeds with a smooth shiny surface, yellowish-brown. Plant grinding develops an intense characteristic smell while the taste is spicy and bitter-burning. The whole plant has a specific lemon smell. The modern use of *S. chinensis* started with a large number of pharmacological and clinical studies conducted in the former USSR in the period 1940−1960 [7,8]. 

Various descriptions of the specific properties of *S. chinensis* are available in English in reviews of Far Eastern medicinal plants. [9]. However, a large amount of information that was reported in Russian journals [4,10,11,12,13,14] is practically not accessible to foreign scientists [15].

More than 40 individual lignans have been reported in the literature, 11 of them, namely schisandrin, gomisin J, gomisin A, gomisin G, angeloygomisin H, angeloygomisin O, schisantherin A, schisantherin B, γ-schisandrin (schisandrin B) and schisandrin C, characterize the *S. chinensis* (Turcz.) Baill. present in several pharmacopeias. It has a chemical composition that differs from the non-pharmacopeia species *Schizandra sphenanthera* Rehd. et Wils [16,17].

**Figure 1:**
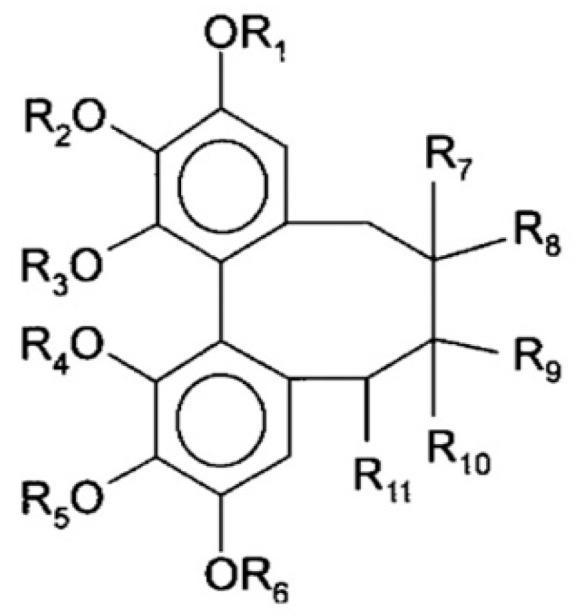
Structure of *S. chinensis* lignans.

Lignans are phenylpropane dimers consisting of two propane residues C_6_–C_3_. Lignans are found in various parts of the plant, especially in the seeds, the underground parts, the wood and woody stems. They may be present in plants in free form and in the form of glycosides [18]. Schizandra lignans are called schisandrins. The chemical skeleton of *S. chinensis* lignans is depicted in Figure 1 and all the substituents are presented in Table 1.

The lignans of *S. chinensis* have been typically extracted with ethanol or hazardous potentially toxic organic solvents such as methanol, chloroform and *n*-hexane. A valid green alternative, in which there is no need to work with a large number of organic solvents and the production does not need explosion-proof rooms, is represented by supercritical fluid extraction (SFE) with many advantages compared to common extraction methods (maceration, percolation Soxhlet extraction) [19,20].

SFE is a green, mild and selective extraction process, one of the best processes to get rid of residual solvent in the extract. Among the supercritical solvents, carbon dioxide is the most common, offering several advantages, because it is non-toxic, non-flammable, cost-effective, environmentally friendly and renewable [25,26,27]. The SFE method is actively studied and applied in the processing of plant materials [28,29].

The lignans of *S. chinensis* were extracted by supercritical CO_2_ (SC-CO_2_) using ethanol as co-solvent [30,31,32]. Different parts of the plant were extracted by SFE, isolating 36 compounds from the leaves, 43 compounds from lignified stems and 36 compounds from rhizomes and roots. *S. chinensis* extracts contain a volatile fraction rich in essential oils (terpenes: monoterpenes, sesquiterpenes; terpenoids: alcohols, esters, ketones) and a non-volatile part (carboxylic acids and lignans). 

## 2. Results and Discussion

**Figure 2:**
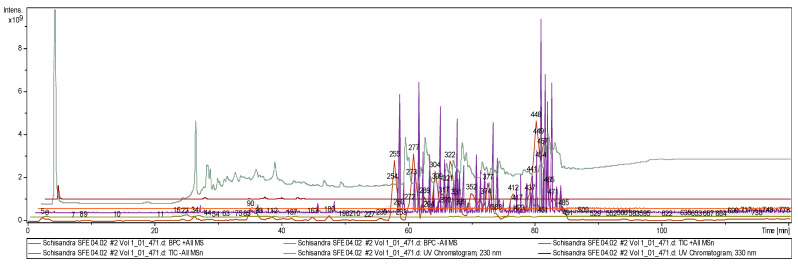
Chemical profiles of *S. chinensis* (Russia), ion chromatogram from SC-CO_2_ extract.

Aiming to optimize the extraction of target analytes from the *S. chinensis* woody liana, several experimental conditions were investigated. Carbon dioxide (CO_2_) was the solvent with the flow rate (10−25 g/min) and 2% ethanol as co-solvent in the liquid phase. Extraction was performed in the pressure range of 200–400 bar and the temperature range of 40–60 °C. The best results were obtained at 350 bar and 60 °C. Increasing the pressure from 350 to 400 bar practically gave no increase in yields. The temperature of 60 °C was chosen as the maximum allowable to avoid the decomposition of target analytes. In this work HPLC-SPD-ESI-MS/MS techniques were used with additional ionization and analysis of fragmented ions. High-accuracy mass spectrometric data were recorded on an ion trap amaZon SL BRUKER DALTONIKS equipped with an ESI source in the mode of negative ions. The three-stage ion separation mode was implemented. Under these conditions a total of 800 peaks were detected in the ion chromatogram (Figure 2). 

Although this approach is not quantitative for evaluating each analyte, it is semiquantitative when comparing a series of extractions and allows better comparison of the yield without loss of individual analytes during fractionation and sample preparation. Only the total extraction yields were completely quantified. 

Table 2 summarizes all the molecular masses of the target analytes isolated from SC-CO_2_ of *S. chinensis*. Among them, 26 biologically active substances were authenticated as lignans (*m*/*z* values and fragment ions) by comparison with literature data [2,22,23,33,34,35,36].

**Figure 3:**
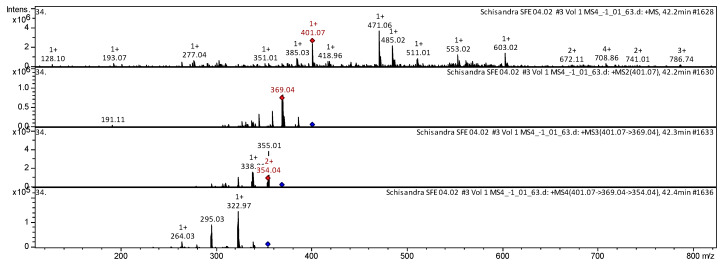
Collision-induced dissociation (CID) spectrum of the schisandrin B (gomisin N, isokadsuranin) from *S. chinensis*, *m*/*z* 401.07.

Figure 3 shows examples of the decoding spectra (collision-induced dissociation (CID) spectrum) of the ion chromatogram obtained using tandem mass spectrometry. The CID spectrum in positive ion modes of schisandrin B (gomisin N, isokadsuranin) from Russian *S. chinensis*.

The [M + H]^+^ ion produced one fragment with *m*/*z* 369.04 (Figure 3). The fragment ion with *m*/*z* 369.04 further formed two daughter ions with *m*/*z* 354.04 and *m*/*z* 338.00. The fragment ion with *m*/*z* 354.04 produced three daughter ions with *m*/*z* 322.97, *m*/*z* 295.03, and *m*/*z* 264.03.

**Figure 4:**
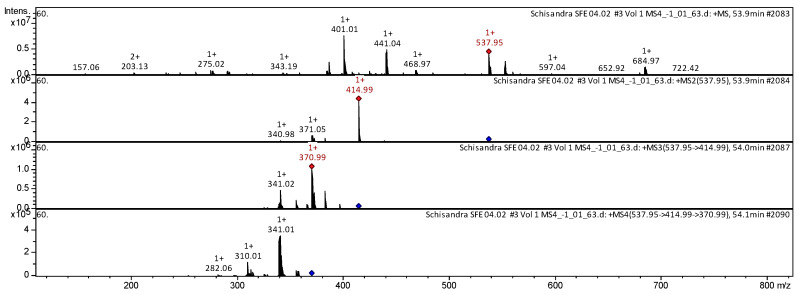
CID spectrum of the schisantherin A (gomisin C) from *S. chinensis*, *m*/*z* 537.95.

The CID spectrum in positive ion modes of schisantherin A (gomisin C) from *S. chinensis* is shown in Figure 4.

The [M + H]^+^ ion produced three fragments with *m*/*z* 414.99, *m*/*z* 371.05 and *m*/*z* 340.98 (Figure 4). The fragment ion with *m*/*z* 414.99 produced two characteristic daughter ions with *m*/*z* 370.99 and *m*/*z* 341.02. The fragment ion with *m*/*z* 370.99 formed three daughter ions with *m*/*z* 341.01, *m*/*z* 310.01, and *m*/*z* 282.06.

**Figure 5:**
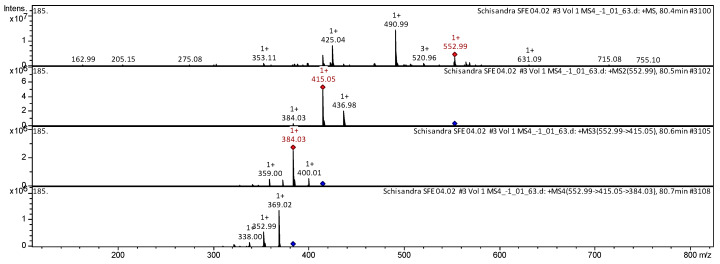
CID spectrum of the benzoylgomisin Q from *S. chinensis*, *m*/*z* 522.99.

The CID spectrum in positive ion modes of benzoylgomisin Q is shown in Figure 5.

The [M + H]^+^ ion produced three fragments with *m*/*z* 415.05, *m*/*z* 436.98 and *m*/*z* 384.03 (Figure 5). The fragment ion with *m*/*z* 415.05 produced three daughter ions with *m*/*z* 400.0, *m*/*z* 384.03 and *m*/*z* 359.00. The fragment ion with *m*/*z* 384.03 yielded three daughter ions with *m*/*z* 369.02, *m*/*z* 352.99, and *m*/*z* 338.00.

## 3. Materials and Methods

### 3.1. Materials

As the objects of the study, samples of *S. chinensis* (woody liana) were purchased from the area of the Peschanka river near Lazovsky district (Sikhote Alin), Primorsky Krai, located at 43°32′ N and 134°33′ E, Russia. All samples were morphologically authenticated according to the current standard of Russian Pharmacopeia [37].

### 3.2. Chemicals and Reagents

HPLC-grade acetonitrile was purchased from Fisher Scientific (Southborough, UK), MS-grade formic acid was from Sigma-Aldrich (Steinheim, Germany). Ultra-pure water was prepared from a SIEMENS ULTRA clear (SIEMENS water technologies, Germany), and all the other chemicals were analytical grade.

### 3.3. SC-CO_2_ Extraction 

SC-CO_2_ extraction was performed using the SFE-500 system (Thar SCF Waters, Milford, USA) supercritical pressure extraction apparatus. System options included co-solvent pump (Thar Waters P-50 High Pressure Pump), for extracting polar samples. CO_2_ flow meter (Siemens, Germany), to measure the amount of CO_2_ being supplied to the system, multiple extraction vessels, to extract different sample sizes or to increase the throughput of the system. Flow rate was 50 mL/min for liquid CO_2_ and 1.76 mL/min for EtOH. Extraction samples of 10 g *Schisandra chinensis* wood were used. The extraction time was counted after reaching the working pressure and equilibrium flow, and it was 6 h for each sample.

### 3.4. Liquid Chromatography

HPLC was performed using Shimadzu LC-20 Prominence HPLC (Shimadzu, Japan), equipped with an UV−VIS detector. The analytical reverse phase column used was a Shodex ODP-40 4E C18 (4.6 × 250 mm, particle size: 4 μm) to perform the separation of multicomponent mixtures. The gradient elution program was as follows: 0.01−4 min, 100% A; 4−60 min, 100−25% A; 60−75 min, 25−0% A; control washing 75−120 min 0% A. The entire HPLC analysis was performed using a UV−VIS detector SPD-20A (Shimadzu, Japan) at wavelengths of 230 and 330 nm, at 17 °C provided with column oven CTO-20A (Shimadzu, Japan) with an injection volume of 20 μL.

### 3.5. Mass Spectrometry

MS analysis was performed on an ion trap amaZon SL (BRUKER DALTONIKS, Germany) equipped with an ESI source in negative ion mode. The optimized parameters were obtained as follows: ionization source temperature: 70 °C, gas flow: 4 L/min, nebulizer gas (atomizer): 7.3 psi, capillary voltage: 4500 V, end plate bend voltage: 1500V, fragmentary: 280 V, collision energy: 60 eV. An ion trap was used in the scan range *m*/*z* 100−1.700 for MS and MS/MS. The capture rate was one spectrum for MS and two spectra for MS/MS. Data collection was controlled by Windows software for BRUKER DALTONIKS. All experiments were repeated three times. A two-stage ion separation mode (MS/MS mode) was implemented.

## 4. Conclusions

An optimized extraction process with SC-CO_2_ (and co-solvent 2% ethanol) of woody liana *S. chinensis* provided the samples for an accurate analytical study by HPLC-SPD-MS/MS techniques. Twenty-six different lignans typical of *S. chinensis* species were identified. This method allows one to get all the studied ligands in a single extract without using a series of approaches and solvents, different in polarity, which not only reduces the environmental pressure, but also simplifies the production process. These data could support future investigations on the quality of pharmaceutical preparations containing these *S. chinensis* extracts. This is because the biological activity is related to the presence of the identified lignans. Their excellent transcutaneous penetration may offer new therapeutic approaches with transdermal preparations based on SC-CO_2_ extracts of *S. chinensis*.

## Figures and Tables

**Table 1:** Chemical structures of *S. chinensis* lignans, according to the authors [18,21,22,23,24].

№	Compound	Formula	R_1_	R_2_	R_3_	R_4_	R_5_	R_6_	R_7_	R_8_	R_9_	R_10_	R_11_
**1**	Schisandrin A (Deoxyschisandrin)	C_24_H_32_O_6_	CH_3_	CH_3_	CH_3_	CH_3_	CH_3_	CH_3_	CH_3_	H	CH_3_	CH_3_	H
**2**	Schisandrol A (Schisandrin)	C_24_H_32_O_7_	CH_3_	CH_3_	CH_3_	CH_3_	CH_3_	CH_3_	CH_3_	H	CH_3_	OH	H
**3**	Schisandrin B (Gomisin N, Isokadsuranin)	C_23_H_28_O_6_	CH_3_	CH_3_	CH_3_	CH_3_	CH_2_		H	CH_3_	H	CH_3_	H
**4**	Schisandrol B (Gomisin A)	C_23_H_28_O_7_	CH_2_		CH_3_	CH_3_	CH_3_	CH_3_	CH_3_	H	CH_3_	OH	H
**5**	Schisandrin C	C_22_H_24_O_6_	CH_2_		CH_3_	CH_3_	CH_3_	CH_3_	H	CH_3_	H	CH_3_	H
**6**	Isoschisandrin	C_24_H_32_O_7_	CH_3_	CH_3_	CH_3_	CH_3_	CH_3_	CH_3_	CH_3_	OH	CH_3_	H	H
**7**	Gomisin K1	C_23_H_30_O_6_	H	CH_3_	CH_3_	CH_3_	CH_3_	CH_3_	H	CH_3_	H	CH_3_	H
**8**	Gomisin K2	C_23_H_30_O_6_	H	CH_3_	CH_3_	CH_3_	CH_3_	CH_3_	CH_3_	H	CH_3_	H	H
**9**	Schisanhenol (Gomisin K3)	C_23_H_30_O_6_	CH_3_	CH_3_	H	CH_3_	CH_3_	CH_3_	CH_3_	H	CH_3_	H	H
**10**	Gomisin H	C_23_H_30_O_7_	CH_3_	CH_3_	H	CH_3_	CH_3_	CH_3_	CH_3_	H	CH_3_	OH	H
**11**	Tigloylgomisin H	C_28_H_36_O_8_	CH_3_	CH_3_	Tigloyl	CH_3_	CH_3_	CH_3_	CH_3_	H	CH_3_	OH	H
**12**	Angeloygomisin H	C_28_H_36_O_8_	CH_3_	CH_3_	Angeloyl	CH_3_	CH_3_	CH_3_	CH_3_	H	CH_3_	OH	H
**13**	Benzoylgomisin H	C_30_H_34_O_8_	CH_3_	CH_3_	Benzoyl	CH_3_	CH_3_	CH_3_	CH_3_	H	CH_3_	OH	H
**4**	Gomisin J	C_22_H_28_O_6_	H	CH_3_	CH_3_	CH_3_	CH_3_	H	H	CH_3_	H	CH_3_	H
**15**	Schisanhenol B	C_22_H_26_O_6_	CH_3_	CH_3_	H	CH_3_	CH_2_		H	CH_3_	H	CH_3_	H
**16**	Gomisin N	C_23_H_28_O_6_	CH_3_	CH_3_	CH_3_	CH_3_	CH_2_		CH_3_	H	CH_3_	H	H
**17**	Gomisin L1	C_22_H_26_O_6_	CH_3_	CH_3_	H	CH_3_	CH_2_		H	CH_3_	H	CH_3_	H
**18**	Gomisin L2	C_22_H_26_O_6_	H	CH_3_	CH_3_	CH_3_	CH_2_		H	CH_3_	H	CH_3_	H
**19**	Gomisin M1	C_22_H_26_O_6_	CH_3_	CH_3_	H	CH_3_	CH_2_		CH_3_	H	CH_3_	H	H
**20**	Gomisin M2	C_22_H_26_O_6_	CH_3_	CH_3_	CH_3_	H	CH_2_		CH_3_	H	CH_3_	H	H
**21**	Gomisin O	C_23_H_28_O_7_	CH_2_		CH_3_	CH_3_	CH_3_	CH_3_	CH_3_	CH_3_	H	H	OH
**22**	Isogomisin O	C_23_H_28_O_7_	CH_3_	CH_3_	CH_3_	CH_3_	CH_2_		H	CH_3_	H	CH_3_	OH
**23**	Angeloylsogomisin O	C_28_H_34_O_8_	CH_2_		CH_3_	CH_3_	CH_3_	CH_3_	CH_3_	CH_3_	H	H	O-angeloyl
**24**	Gomisin P	C_23_H_28_O_8_	CH_2_		CH_3_	CH_3_	CH_3_	CH_3_	H	CH_3_	OH	CH_3_	OH
**25**	Tigloylgomisin P	C_28_H_34_O_9_	CH_2_		CH_3_	CH_3_	CH_3_	CH_3_	H	CH_3_	OH	CH_3_	O-tigloyl
**26**	Angeloylgomisin P (Schisantherin C)	C_28_H_34_O_9_	CH_2_		CH_3_	CH_3_	CH_3_	CH_3_	H	CH_3_	OH	CH_3_	O-angeloyl
**27**	Schisantherin A (Gomisin C)	C_30_H_32_O_9_	CH_2_		CH_3_	CH_3_	CH_3_	CH_3_	CH_3_	CH_3_	OH	H	O-bensoyl
**28**	Schisantherin B (Gomisin B, Schisandrer B)	C_28_H_34_O_9_	CH_2_		CH_3_	CH_3_	CH_3_	CH_3_	CH_3_	CH_3_	OH	H	O-angeloyl
**29**	Gomisin S	C_23_H_30_O_7_	CH_3_	CH_3_	CH_3_	CH_3_	CH_3_	CH_3_	H	CH_3_	H	CH_3_	OH
**30**	Gomisin R (6-Epi-gomisin)	C_22_H_24_O_7_	CH_2_		CH_3_	CH_3_	CH_2_		CH_3_	H	CH_3_	H	H
**31**	Deangeloylgomisin B	C_23_H_28_O_8_	CH_2_		CH_3_	CH_3_	CH_3_	CH_3_	CH_3_	CH_3_	OH	H	OH
**32**	Gomisin F	C_28_H_34_O_9_	CH_3_	CH_3_	CH_3_	CH_3_	CH_2_		CH_3_	CH_3_	OH	H	O-angeloyl
**33**	Gomisin G	C_30_H_32_O_9_	CH_3_	CH_3_	CH_3_	CH_3_	CH_2_		CH_3_	CH_3_	OH	H	O-bensoyl
**34**	Epigomisin O	C_23_H_28_O_7_	CH_2_		CH_3_	CH_3_	CH_3_	CH_3_	CH_3_	CH_3_	CH_3_	H	H
**35**	Angeloylgomisin Q	C_29_H_38_O_9_	CH_3_	CH_3_	CH_3_	CH_3_	CH_3_	CH_3_	H	CH_3_	CH_3_	OH	O-angeloyl

**Table 2:** Lignans from *S. chinensis* SC-CO_2_ extract.

№	Identification	Formula	Calcula-ted Mass	Observed Mass [M + H]^+^	Observed Mass [M + Na]^+^	MS/MS Stage 1 Fragmentation	MS/MS Stage 2 Fragmentati-on	MS/MS Stage 3 Fragmentation
**1**	**Schisandrin C** [(12S,13R)-3,22-dimethoxy-12,13-dimethyl-5,7,18,20-tetraoxapentacyclo [13.7.0.02,10.04,8.017,21]docosa-1(22),2,4(8),9,15,17(21)-hexaene]	C_22_H_24_O_6_	384.4224	385.02		355.01; 323.02	323.01; 299.02; 269.03; 234.98	307.98; 235.05
**2**	**Gomisin M1 (Gomisin L1)** [(9S,10R)-4,5,19-trimethoxy-9,10-dimethyl-15,17-dioxatetracyclo [10.7.0.02,7.014,18]nonadeca-1(19),2,4,6,12,14(18)-hexaen-3-ol]	C_22_H_26_O_6_	386.4382		408.95	290.99; 394.03; 326.08; 274.96	260.97; 242.89; 172.97	
**3**	**Gomisin L2** [(9S,10R)-3,4,19-trimethoxy-9,10-dimethyl-15,17-dioxatetracyclo[10.7.0.02,7.014,18]nonadeca-1(19),2,4,6,12,14(18)-hexaen-5-ol]	C_22_H_26_O_6_	386.4382	386.98		356.98; 325.00; 284.93; 259.03; 226.99; 167.02; 137.17	297.04; 226.98; 182.97	
**4**	**Gomisin M2** [(9S,10R)-3,4,5-trimethoxy-9,10-dimethyl-15,17-dioxatetracyclo[10.7.0.02,7.014,18]nonadeca-1(19),2,4,6,12,14(18)-hexaen-19-ol]	C_22_H_26_O_6_	386.4382	387.01		355.01; 324.01; 284.97	339.98; 324.02; 284.97; 226.96	324.94; 296.90
**5**	**Gomisin J** [(9S,10R)-3,4,15,16-tetramethoxy-9,10-dimethyltricyclo[10.4.0.02,7]hexadeca-1(16),2,4,6,12,14-hexaene-5,14-diol]	C_22_H_28_O_6_	388.4541	389.04		325.03; 357.01; 226.96; 286.97	227.01; 241.00; 269.03; 297.01	226.98; 198.98
**6**	**Pregomisin** [5-[(2S,3R)-4-(3-hydroxy-4,5-dimethoxyphenyl)-2,3-dimethylbutyl]-2,3-dimethoxyphenol]	C_22_H_30_O_6_	390.47	391.00		237.07; 205.03; 288.91; 326.96; 359.00	205.00; 173.00	
**7**	**Schisandrin B (Gomisin N, Isokadsuranin)** [3,4,5,19-tetramethoxy-9,10-dimethyl-15,17-dioxatetracyclo[10.7.0.02,7.014,18]nonadeca-1(19),2,4,6,12,14(18)-hexaene]	C_23_H_28_O_6_	400.3648	401.07		369.04	354.04; 338.00	322.97; 295.03; 264.03
**8**	**Schisanhenol (Gomisin K3)** [(9S,10R)-4,5,14,15,16-pentamethoxy-9,10-dimethyltricyclo[10.4.0.02,7]hexadeca-1(16),2,4,6,12,14-hexaen-3-ol]	C_23_H_30_O_6_	402.4807	403.05		371.01; 340.03; 301.01; 259.00	340.03; 315.01; 300.98; 286.01; 233.07	324.99; 270.99; 227.02
**9**	**Gomisin O** [(8R,9S,10S)-3,4,5,19-tetramethoxy-9,10-dimethyl-15,17-dioxatetracyclo[10.7.0.02,7.014,18]nonadeca-1(19),2,4,6,12,14(18)-hexaen-8-ol]	C_23_H_28_O_7_	416.3642	417.01		356.97; 373.01	329.00	313.97; 270.00
**10**	**Erigomisin O** [(8S,9S,10S)-3,4,5,19-tetramethoxy-9,10-dimethyl-15,17-dioxatetracyclo[10.7.0.02,7.014,18]nonadeca-1(19),2,4,6,12,14(18)-hexaen-8-ol]	C_23_H_28_O_7_	416.3642	416.96		356.98; 340.98; 308.97	328.95; 313.98	
**11**	**Schisandrin A (Deoxyschisandrin)** [(9R,10S)-3,4,5,14,15,16-hexamethoxy-9,10-dimethyltricyclo[10.4.0.02,7]hexadeca-1(16),2,4,6,12,14-hexaene]	C_24_H_32_O_6_	416.5073	417.01		316.00; 346.99; 402.01	300.96; 284.95; 242.02	
**12**	Demethylated metabolites of Schisandrol A				440.95	279.00; 322.89; 306.86; 258.89; 202.99	259.94;220.86; 137.02	
**13**	**Schisandrol A (Schisandrin)** [(9R,10S)-3,4,5,14,15,16-hexamethoxy-9,10-dimethyltricyclo[10.4.0.02,7]hexadeca-1(16),2,4,6,12,14-hexaen-9-ol]	C_24_H_32_O_7_	432.5067	433.50		415.03	384.04; 359.03	368.99; 353.05
**14**	**7, 8-Dihydroxy-schisandrin**	C_24_H_32_O_8_	448.5061		470.95	332.90; 348.90; 200.84; 230.30; 274.74	332.89; 274.94; 244.93; 202.98; 155.17	
**15**	**Tigloylgomisin O** [[(8R,9S,10S)-3,4,5,19-tetramethoxy-9,10-dimethyl-15,17-dioxatetracyclo[10.7.0.02,7.014,18]nonadeca-1(19),2,4,6,12,14(18)-hexaen-8-yl] (E)-2-methylbut-2-enoate]	C_28_H_34_O_8_	498.5648		521.92	208.01; 250.08; 304.99; 359.99; 402.85; 436.83	191.00; 375.89	
**16**	**Angeloylsogomisin O** [[(9S,10S,11R)-3,4,5,19-tetramethoxy-9,10-dimethyl-15,17-dioxatetracyclo[10.7.0.02,7.014,18]nonadeca-1(19),2,4,6,12,14(18)-hexaen-11-yl] (Z)-2-methylbut-2-enoate]	C_28_H_34_O_8_	498.5648	387.16		355.12	323.00; 341.00; 295.02; 262.94; 210.100	308.98; 262.97; 220.24
**17**	**Angeloygomisin H** [[(9S,10S)-10-hydroxy-4,5,14,15,16-pentamethoxy-9,10-dimethyl-3-tricyclo[10.4.0.02,7]hexadeca-1(16),2,4,6,12,14-hexaenyl] (Z)-2-methylbut-2-enoate]	C_28_H_36_O_8_	500.3806	500.95		368.93; 433.87; 472.83; 334.94; 288.84; 244.92; 207.21; 169.02	368.92; 352.97; 299.90; 244.80; 208.95; 156.99; 125.89	
**18**	**Micrantherin A**	C_28_H_36_O_8_	500.5806	522.93		422.91; 328.94; 386.00; 476.94	407.87; 392.92; 364.93; 350.88; 320.91; 295.02	
**19**	**Gomisin E** [(11R,12R,15R,24S,25S)-12-hydroxy-18,19,20-trimethoxy-11,12,24,25-tetramethyl-4,6,9,14-tetraoxapentacyclo[13.7.3.03,7.08,22.016,21]pentacosa-1,3(7),8(22),16,18,20-hexaen-13-one]	C_28_H_34_O_9_	514.3642	514.99		384.98; 355.03; 322.99	354.99; 322.97	
**20**	**Schisantherin D** [[(11S,12S,13S)-12-hydroxy-3,22-dimethoxy-12,13-dimethyl-5,7,18,20-tetraoxapentacyclo[13.7.0.02,10.04,8.017,21]docosa-1(22),2,4(8),9,15,17(21)-hexaen-11-yl]	C_29_H_28_O_9_	520.5272		542.89	380.89; 408.36; 451.55; 334.99; 200.93		
**21**	**Benzoylgomisin O** [[(8R,9S,10S)-3,4,5,19-tetramethoxy-9,10-dimethyl-15,17-dioxatetracyclo[10.7.0.02,7.014,18]nonadeca-1(19),2,4,6,12,14(18)-hexaen-8-yl] benzoate]	C_30_H_32_O_9_	520.5703		542.91	380.89	364.66; 308.93; 193.02	
**22**	**Benzoylgomisin H** [[(9S,10S)-10-hydroxy-4,5,14,15,16-pentamethoxy-9,10-dimethyl-3-tricyclo[10.4.0.02,7]hexadeca-1(16),2,4,6,12,14-hexaenyl] benzoate]	C_30_H_34_O_8_	522.5862	522.99		491.30; 448.09; 421.07; 399.03; 377.05; 335.11; 302.95; 269.78	271.39; 213.02	
**23**	**Gomisin D** [12,25-dihydroxy-18,19,20-trimethoxy-11,12,24,25-tetramethyl-4,6,9,14-tetraoxapentacyclo[13.7.3.03,7.08,22.016,21]pentacosa-1,3(7),8(22),16,18,20-hexaen-13-one]	C_28_H_34_O_10_	530.5636		553.97	510.97; 478.98; 400.97; 372.91; 334.94; 248.99; 202.87	382.92; 354.95; 339.03; 312.11; 277.00; 248.99; 189.03	
**24**	**Gomisin G** [[(9S,10S,11S)-10-hydroxy-3,4,5,19-tetramethoxy-9,10-dimethyl-15,17-dioxatetracyclo[10.7.0.02,7.014,18]nonadeca-1(19),2,4,6,12,14(18)-hexaen-11-yl] benzoate]	C_30_H_32_O_9_	536.3697	536.93		436.92; 414.99; 371.03; 341.04	422.80; 390.84; 360.99	
**25**	**Schisantherin A (Gomisin C)** [[(8S,9S,10S)-9-hydroxy-3,4,5,19-tetramethoxy-9,10-dimethyl-15,17-dioxatetracyclo[10.7.0.02,7.014,18]nonadeca-1(19),2,4,6,12,14(18)-hexaen-8-yl] benzoate]	C_30_H_32_O_9_	536.5697	537.95		414.99; 371.05; 340.98	370.99; 341.02	341.01; 310.01; 282.06
**26**	**Benzoylgomisin Q** [[(8S,9S,10S)-9-hydroxy-3,4,5,14,15,16-hexamethoxy-9,10-dimethyl-8-tricyclo[10.4.0.02,7]hexadeca-1(16),2,4,6,12,14-hexaenyl] benzoate]	C_31_H_36_O_9_	552.3121	552.99		415.05; 436.98; 384.03	384.03; 400.01; 359.00	369.02; 352.99; 338.00
